# Effect of human recreation on bird anti-predatory response

**DOI:** 10.7717/peerj.5093

**Published:** 2018-06-21

**Authors:** Yves Bötsch, Selina Gugelmann, Zulima Tablado, Lukas Jenni

**Affiliations:** 1 Swiss Ornithological Institute, Sempach, Switzerland; 2 Institute of Evolutionary Biology and Environmental Studies, University of Zurich, Zurich, Switzerland; 3 ETH Zurich, Zurich, Switzerland

**Keywords:** Human disturbance, Flush distance, Habituation, Escape distance

## Abstract

Wildlife perceive humans as predators, and therefore normally flushes. Flight initiation distance (FID) is the distance a human can approach an animal at a steady pace until it flushes. Recently, several studies showed differences in within-species FID according to human presence by comparing urban and rural habitats, with urban birds showing reduced FIDs. However, urban and rural habitats also differ in structure, which might affect FID. Therefore, in order to understand the real effect of human presence, we investigated whether differences in FID are also present in natural habitats (forests), differing only in the intensity of human use for recreation. We found that human frequentation had a distinct effect on bird escape responses, with shorter FIDs in forests more-heavily frequented by humans than in forests rarely visited by humans. Whether this finding is driven by non-random spatial distribution of personalities (shy vs. bold) or phenotypic plasticity (habituation to humans) cannot be assessed with our data. Studies relying on FIDs should also incorporate human recreation intensity, as this affects the measurements strongly.

## Introduction

Human disturbance through recreational activities has been found to negatively affect wildlife (Thiel et al., 2007; Kangas et al., 2010; Coppes et al., 2017; Bötsch, Tablado & Jenni, 2017). To protect wild animals from the negative effects of recreation, zones with restricted access or buffer zones around breeding areas are a widely recommended mitigation measure (Rodgers & Smith, 1995; Fernández-Juricic, Jimenez & Lucas, 2001; Blumstein et al., 2003; Ikuta & Blumstein, 2003; Webb & Blumstein, 2005). In order to define these buffer zones an appropriate set-back distance, matching the focal species needs, has to be chosen. Unfortunately, conservation measures often have to be defined quickly and ad hoc, without time for area-specific in-depth studies. Often the so-called flight initiation distance (FID) has been used to define a minimal set-back distance. FID is the distance at which humans can approach a species before triggering its anti-predatory/escape behavior. FIDs have been used widely for decades and still are considered a good surrogate for set-back distances (Samia et al., 2017).

Many modulating factors have been found to affect these FIDs (Gutzwiller & Marcum, 1997; Eason et al., 2006; Thiel et al., 2007; Randler, 2008; Carrete & Tella, 2010; Legagneux & Ducatez, 2013; Cavalli et al., 2016; Wilson-Aggarwal et al., 2016; Lethlean et al., 2017). There are intrinsic differences between species and individuals (Blumstein et al., 2003; Carrete & Tella, 2010), but also many habitat- and context-specific effects have been reported, such as shorter FIDs in denser habitats (Tablado & Jenni, 2017). Recently, studies have shown differences in bird tolerance to human approach across an urban–rural gradient, with urban populations showing reduced FIDs compared to rural conspecifics (McGiffin et al., 2013; Møller et al., 2013; Cavalli et al., 2016). These reduced FIDs have been attributed to habituation to human presence and/or the selection of human-tolerant individuals (personalities) in urban environments (McGiffin et al., 2013; Cavalli et al., 2016; Vincze et al., 2016; Samia et al., 2017; Sprau & Dingemanse, 2017). However, urban and rural habitats do not only differ in the presence of humans but also in habitat structure which might also affect bird escape reactions (FID; Whittingham et al., 2004; Thiel et al., 2007). It remains then unclear whether to what extent the differences in FIDs between urban and rural environments are due to differences between habitats or to the frequency of humans. Moreover, little is known about the effect of lower levels of human presence, such as that occurring during recreation in natural areas, on bird anti-predatory behavior.

Therefore, the aim of this study was to investigate the effect of human presence per se on anti-predatory responses in natural areas (forests). That is, whether FIDs of birds in forests near large urban settlements, and thus often frequented by recreationists, differ from bird FIDs in rarely visited forests of similar structure.

## Material and Methods

### Study sites

We measured FIDs in three different forests varying in the distance to cities and thus, on the level of recreation they hold. “Allschwilerwald” near Basel (193,000 inhabitants), Switzerland, 47°32′N 7°32′E, “Sihlwald” close to Zurich (415,000 inhabitants), Switzerland, 47°16′N 8°33′E and “Forêt de Chaux,” Département du Bourgogne-Franche-Comté, France, 47°5′N 5°41′E, which has no big cities nearby (being Besançon the closest city with 116,000 inhabitants) and therefore human recreation occurred at a very low level. The two forests near cities were on average frequented by 20 people per hour, while we detected no more than one human passage per day during our daily work in the Forêt de Chaux. These forests were all fully free to access for people. In 2015 we measured FIDs in the “Forêt de Chaux” (11 March until 11 April), and in 2016 we measured FIDs in all three forests (13 March until 18 April).

All three forests were deciduous. Allschwilerwald and Forêt de Chaux were both dominated by pedunculate oak (*Quercus robur*), and Sihlwald by European beech (*Fagus sylvatica*). All three forests were closed-canopy mature forests with large dominating trees and only a sparse shrub layer of similar density (see Fig. S1). Conifers were rare. During the non-foliated season (when we measured FIDs, dates mentioned above), visibility was high and unobstructed by vegetation (except for tree stems), and birds were easily detected at ranges from 60 up to 100 m in all forests.

### FID-measures

As the vegetation density affects visibility (both bird detection by humans but also detection of humans by birds), and therefore also FID, we took FID measures only in early spring, from the beginning of March until mid-April. In this way, we avoided the foliated season and obtained FID measures that were independent of vegetation density. By measuring FIDs only in these two months we were also reducing the variation in anti-predatory-responses across life-history stages. To obtain a sufficient sample size we focused on a few common species, which were all abundant at all sites: Common Blackbird (*Turdus merula*), Common Chaffinch (*Fringilla coelebs*), Eurasian Nuthatch (*Sitta europaea*), European Robin (*Erithacus rubecula*), Great Tit (*Parus major*), Marsh Tit (*Poecile palustris*), Short-toed Treecreeper (*Certhia brachydactyla*), Song Thrush (*Turdus philomelos*), and Winter Wren (*Troglodytes troglodytes*).

Flight initiation distance was measured by a single person approaching a located bird, that did not yet react visibly to our presence (e.g., by alert posture or alarm calling), at steady pace (1 m/s). Only single birds, either singing, feeding or resting were measured, as birds on a nest or birds in groups have been shown to flush differently (Fernández-Juricic, Jimenez & Lucas, 2002). FID was measured with range finding binoculars (ZEISS Victory 10×45 T* RF) as the horizontal line between the observer and the tree, bush or ground where the bird was before flushing. We also measured the height above ground where the bird was sitting as well as the distance at which the measuring person discovered the individual (starting distance) (Blumstein, 2003).

### Data analysis

We applied a general linear mixed model to analyze the FID measures using the lme4-package in R v. 3.3.0 (Bates et al., 2015; R Core Team, 2016). We included as explanatory variables: starting distance, height above ground, time of day (daytime), Julian date, species, average distance from FID-measurement points to closest city border (city was defined after the OECD-EC definition of at least 50,000 inhabitants), and the interaction between species and distance. The effect sizes of the interactions describe the change in FID with increasing distance from a city within a species (Table 1). Most continuous variables (when necessary) were standardized to facilitate model convergence (mean = 0, sd = 1). We included observer nested within forest, nested within year as random factors to account for variability among years and forests and to account for the non-independence of the measures taken by the different observers. To account for phylogenetic relatedness between the species, we included the family of each species as additional random factor into the model. We visually checked for the goodness of fit by plotting residuals.

**Table 1:** Model output of the FID-model (General linear mixed model, fit by restricted maximum likelihood (REML)) with the estimates and the corresponding 95% credible intervals (CrI).

Variable	Estimate	95% CrI
Intercept	6.77	0.28; 13.27
Starting distance	7.03	6.48; 7.58
Height	−2.56	−3.11; −2.01
Time	−0.24	−0.73; 0.25
Julian date	−0.08	−0.56; 0.41
Distance (km)	0.71	0.45; 0.96
Common Chaffinch (CC)	1.89	−5.76; 9.77
Short-toed Treecreeper (StT)	−2.40	−10.95; 6.24
Eurasian Nuthatch (EN)	1.07	−6.90; 9.11
Great Tit (GT)	3.14	−4.76; 10.93
European Robin (ER)	0.85	−6.92; 8.64
Song Thrush (ST)	1.80	−2.87; 6.37
Marsh Tit (MT)	3.94	−4.42; 12.06
Winter Wren (WW)	1.73	−6.14; 9.85
Distance (km) × Species (CC)	−0.42	−0.64; −0.20
Distance (km) × Species (StT)	−0.47	−0.76; −0.17
Distance (km) × Species (EN)	−0.57	−0.81; −0.33
Distance (km) × Species (GT)	−0.65	−0.87; −0.44
Distance (km) × Species (ER)	−0.45	−0.68; −0.23
Distance (km) × Species (ST)	−0.57	−0.88; −0.26
Distance (km) × Species (MT)	−0.71	−0.96; −0.45
Distance (km) × Species (WW)	−0.60	−0.83; −0.37

**Note:**

The reference category is the Common Blackbird.

For making inference we used a Bayesian approach (after Korner-Nievergelt et al., 2015). We simulated 10,000 random samples from the posterior distribution by using the sim-function from the R-package arm (Gelman & Su, 2015). From these random samples we used the 2.5% and 97.5% quantiles as the limits of the 95% credible interval. To be able to investigate species-specific differences in relation to the level of recreation (approximated by the distance from cities), we computed posterior probabilities (PP). PPs describe the probability that, for a given species, FID actually increases with distance from cities (i.e., slope differs from zero). PPs can take values between 0.5 and 1, with larger values representing a larger effect of distance.

## Results

For each species we had the following FID sample sizes: 73 for Common Blackbird, 121 for Common Chaffinch, 62 for Eurasian Nuthatch, 94 for European Robin, 132 for Great Tit, 39 for Marsh Tit, 23 for Short-toed Treecreeper, 26 for Song Thrush, and 74 for Winter Wren.

**Figure 1:**
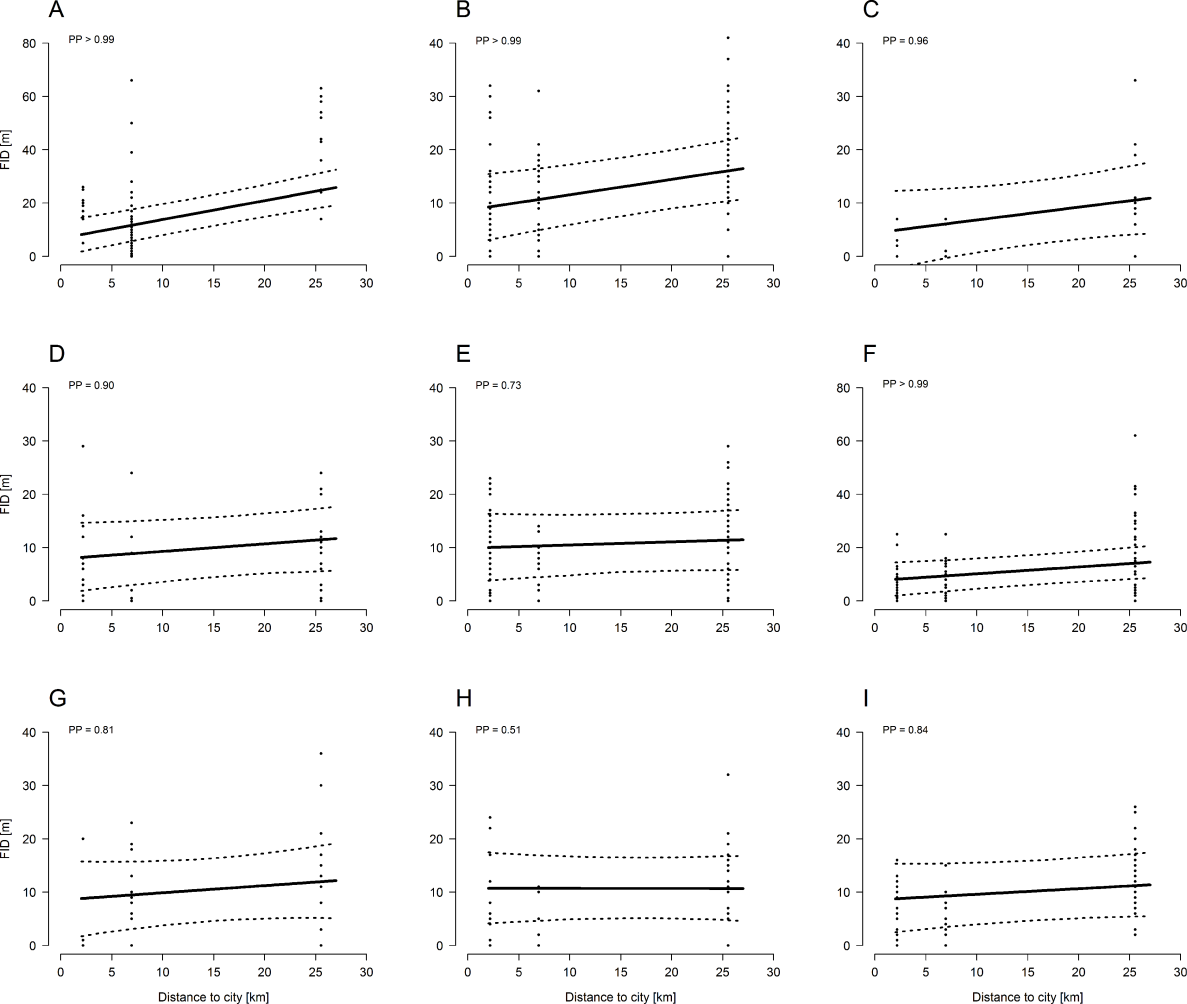
Model estimates (± 95 CrI, dashed lines) of species-specific flight-initiation distances of nine common forest bird species in relation to recreation intensity (approximated by distance from the nearest city). Forests far away from cities are rarely frequented by humans whereas forests close to cities are highly frequented by humans. Black dots represent the FID measures. PP: posterior probability. The larger the PP the stronger is the effect of recreation on FID. (A) Common Blackbird, (B) Common Chaffinch, (C) Short-toed Treecreeper, (D) Eurasian Nuthatch, (E) Great Tit, (F) European Robin, (G) Song Thrush, (H) Marsh Tit, and (I) Winter Wren.

All bird species, except the marsh tit, showed a trend towards larger FIDs in forests further away from a city (Fig. 1; Table 1). Considering the PP (Fig. 1) of the regression line slopes, the effect of recreation, approximated as distance to cities, on FID was especially strong in Common Blackbirds, Common Chaffinch, European Robin, Short-toed Treecreeper, and Eurasian Nuthatch.

Apart from distance to cities, other factors that also seemed to determine FID were height above ground and starting distance. The higher above ground a bird was located, the shorter was its horizontal FID (Table 1), whereas the starting distance was positively correlated with the FID (Table 1).

## Discussion

Recreation showed a substantial effect on bird anti-predatory responses. Birds had shorter FIDs in forests close to cities (intensively frequented by humans) than in forests further away from cities (i.e., less visited by humans). Because the habitat was very similar, this indicates that the shorter FID is most likely due to human presence. It also agrees with the studies suggesting that the shorter FIDs found in urban environments, when compared to rural ones, are mainly due to human presence (Cavalli et al., 2016). People may be perceived by naive wildlife as predators (Frid & Dill, 2002; Beale & Monaghan, 2004). This may explain why we find large FIDs in forests where birds did not have that much experience with humans (“non-habituated”). In contrast, in forests with higher human frequentation birds could have “habituated” to non-threatening recreationists and reduce their behavioral anti-predatory response. An alternative explanation for our finding could be a redistribution of personalities, as recently shown by Sprau & Dingemanse (2017). That is, personality types could be non-randomly distributed along a gradient with bolder animals being more frequent in forests with more human presence (closer to cities), whereas shyer individuals would be restricted to less frequently visited forests. We also expect that habituation will occur faster or slower depending on the species and the personality of the individuals. Further studies may investigate whether there is a threshold of pedestrians required for birds to become habituated or rearranged according to their personality, resulting in different escape strategy patterns.

Whether non-random settlement or phenotypic plasticity is the driving factor for reduced FIDs in heavily frequented forests cannot be inferred from our data. We acknowledge that with only three forests along a distance-to-the-city gradient, it is hard to generalize and we cannot rule out completely the influences of other local factors. However, the proximity of the three forests (i.e., similar climatic conditions and socio-economic areas), the fact that the species are the same and the similarity in vegetation structure among the three forests, strongly suggests that the effect is most likely due to differences in exposure to humans.

## Conclusion

In conclusion, bird anti-predatory responses (FID) are affected by human recreation intensity. Thus, when FID is to be used for defining set-back distances, local FIDs should be measured to avoid mismatching between bird tolerances to humans in areas where the FID was measured and the ones in the local area where it will be applied. If human disturbance already occurred at a given site, the most vulnerable species or individuals might already have left the site. Therefore, FIDs always have to be carefully interpreted before using it to establish set-back distances. Our finding implies that future studies measuring FIDs should also incorporate site-specific human recreation intensity as a modulating factor for FIDs.

## Supplemental Information

10.7717/peerj.5093/supp-1 Supplemental Information 1 Fig. S1. Vegetation measures (mean model estimates with 95% credible intervals) describing the three different forests where FID measures have been taken. Note that vegetation measures were taken within the framework of a different study and thus do not entirely coincide in season or exact locations within forest with the FID measures. Therefore we could not directly include these measures in the model, but they still show that the forests were comparable in terms of habitat structure. There are differences in ground cover between certain forests. However, these differences are not likely to affect FIDs since our FID measures are taken in early spring (non-foliated season) and FIDs are usually more affected by higher-layers of vegetation (i.e. shrub and canopy layers). Aw: Allschwilerwald, Sw: Sihlwald, FdC: Forêt de Chaux.

10.7717/peerj.5093/supp-2 Supplemental Information 2 Raw data.

## Additional Information and Declarations

### Data Availability

The following information was supplied regarding data availability: The raw data are provided in the Supplemental File.

## References

[ref-1] Bates D, Mächler M, Bolker B, Walker S (2015). Fitting linear mixed-effects models using lme4. Journal of Statistical Software.

[ref-2] Beale CM, Monaghan P (2004). Human disturbance: people as predation-free predators?. Journal of Applied Ecology.

[ref-3] Blumstein DT (2003). Flight-initiation distance in birds is dependent on intruder starting distance. Journal of Wildlife Management.

[ref-4] Blumstein DT, Anthony LL, Harcourt R, Ross G (2003). Testing a key assumption of wildlife buffer zones: is flight initiation distance a species-specific trait?. Biological Conservation.

[ref-5] Bötsch Y, Tablado Z, Jenni L (2017). Experimental evidence of human recreational disturbance effects on bird-territory establishment. Proceedings of the Royal Society B: Biological Sciences.

[ref-6] Carrete M, Tella JL (2010). Individual consistency in flight initiation distances in burrowing owls: a new hypothesis on disturbance-induced habitat selection. Biology Letters.

[ref-7] Cavalli M, Baladrón AV, Isacch JP, Biondi LM, Bó MS (2016). Differential risk perception of rural and urban Burrowing Owls exposed to humans and dogs. Behavioural Processes.

[ref-8] Coppes J, Burghardt F, Hagen R, Suchant R, Braunisch V (2017). Human recreation affects spatio-temporal habitat use patterns in red deer (*Cervus elaphus*). PLOS ONE.

[ref-9] Eason PK, Sherman PT, Rankin O, Coleman B (2006). Factors affecting flight initiation distance in American robins. Journal of Wildlife Management.

[ref-10] Fernández-Juricic E, Jimenez MD, Lucas E (2001). Alert distance as an alternative measure of bird tolerance to human disturbance: implications for park design. Environmental Conservation.

[ref-11] Fernández-Juricic E, Jimenez MD, Lucas E (2002). Factors affecting intra- and inter-specific variations in the difference between alert distances and flight distances for birds in forested habitats. Canadian Journal of Zoology.

[ref-12] Frid A, Dill L (2002). Human-caused disturbance stimuli as a form of predation risk. Conservation Ecology.

[ref-13] Gelman A, Su Y-S (2015). https://CRAN.R-project.org/package=arm.

[ref-14] Gutzwiller KJ, Marcum HA (1997). Bird reactions to observer clothing color: implications for distance-sampling techniques. Journal of Wildlife Management.

[ref-15] Ikuta LA, Blumstein DT (2003). Do fences protect birds from human disturbance?. Biological Conservation.

[ref-16] Kangas K, Luoto M, Ihantola A, Tomppo E, Siikamäki P (2010). Recreation-induced changes in boreal bird communities in protected areas. Ecological Applications.

[ref-17] Korner-Nievergelt F, Roth T, von Felten S, Guélat J, Almasi B, Korner-Nievergelt P (2015). Bayesian Data Analysis in Ecology Using Linear Models with R, BUGS, and Stan.

[ref-18] Legagneux P, Ducatez S (2013). European birds adjust their flight initiation distance to road speed limits. Biology Letters.

[ref-19] Lethlean H, Van Dongen WFD, Kostoglou K, Guay P-J, Weston MA (2017). Joggers cause greater avian disturbance than walkers. Landscape and Urban Planning.

[ref-20] McGiffin A, Lill A, Beckman J, Johnstone CP (2013). Tolerance of human approaches by Common Mynas along an urban-rural gradient. Emu–Austral Ornithology.

[ref-21] Møller AP, Grim T, Ibáñez-Álamo JD, Markó G, Tryjanowski P (2013). Change in flight initiation distance between urban and rural habitats following a cold winter. Behavioral Ecology.

[ref-22] Randler C (2008). Risk assessment by crow phenotypes in a hybrid zone. Journal of Ethology.

[ref-23] R Core Team (2016). R: A Language and Environment for Statistical Computing.

[ref-24] Rodgers JA, Smith HT (1995). Set-back distances to protect nesting bird colonies from human disturbance in Florida. Conservation Biology.

[ref-25] Samia DSM, Blumstein DT, Diaz M, Grim T, Ibáñez-Álamo JD, Jokimäki J, Tätte K, Markó G, Tryjanowski P, Møller AP (2017). Rural-urban differences in escape behavior of European birds across a latitudinal gradient. Frontiers in Ecology and Evolution.

[ref-26] Sprau P, Dingemanse NJ (2017). An approach to distinguish between plasticity and non-random distributions of behavioral types along urban gradients in a wild passerine bird. Frontiers in Ecology and Evolution.

[ref-27] Tablado Z, Jenni L (2017). Determinants of uncertainty in wildlife responses to human disturbance. Biological Reviews.

[ref-28] Thiel D, Ménoni E, Brenot J-F, Jenni L (2007). Effects of recreation and hunting on flushing distance of capercaillie. Journal of Wildlife Management.

[ref-29] Vincze E, Papp S, Preiszner B, Seress G, Bókony V, Liker A (2016). Habituation to human disturbance is faster in urban than rural house sparrows. Behavioral Ecology.

[ref-30] Webb NV, Blumstein DT (2005). Variation in human disturbance differentially affects predation risk assessment in Western Gulls. Condor.

[ref-31] Whittingham MJ, Butler SJ, Quinn JL, Cresswell W (2004). The effect of limited visibility on vigilance behaviour and speed of predator detection: implications for the conservation of granivorous passerines. Oikos.

[ref-32] Wilson-Aggarwal JK, Troscianko JT, Stevens M, Spottiswoode CN (2016). Escape distance in ground-nesting birds differs with individual level of camouflage. American Naturalist.

