# Supplementary figures and images for: Effect of human recreation on bird anti-predatory response
Source: PeerJ. 2018 Jun 21;6:e5093. doi: 10.7717/peerj.5093 (PMC6015756; doi:10.7717/peerj.5093)

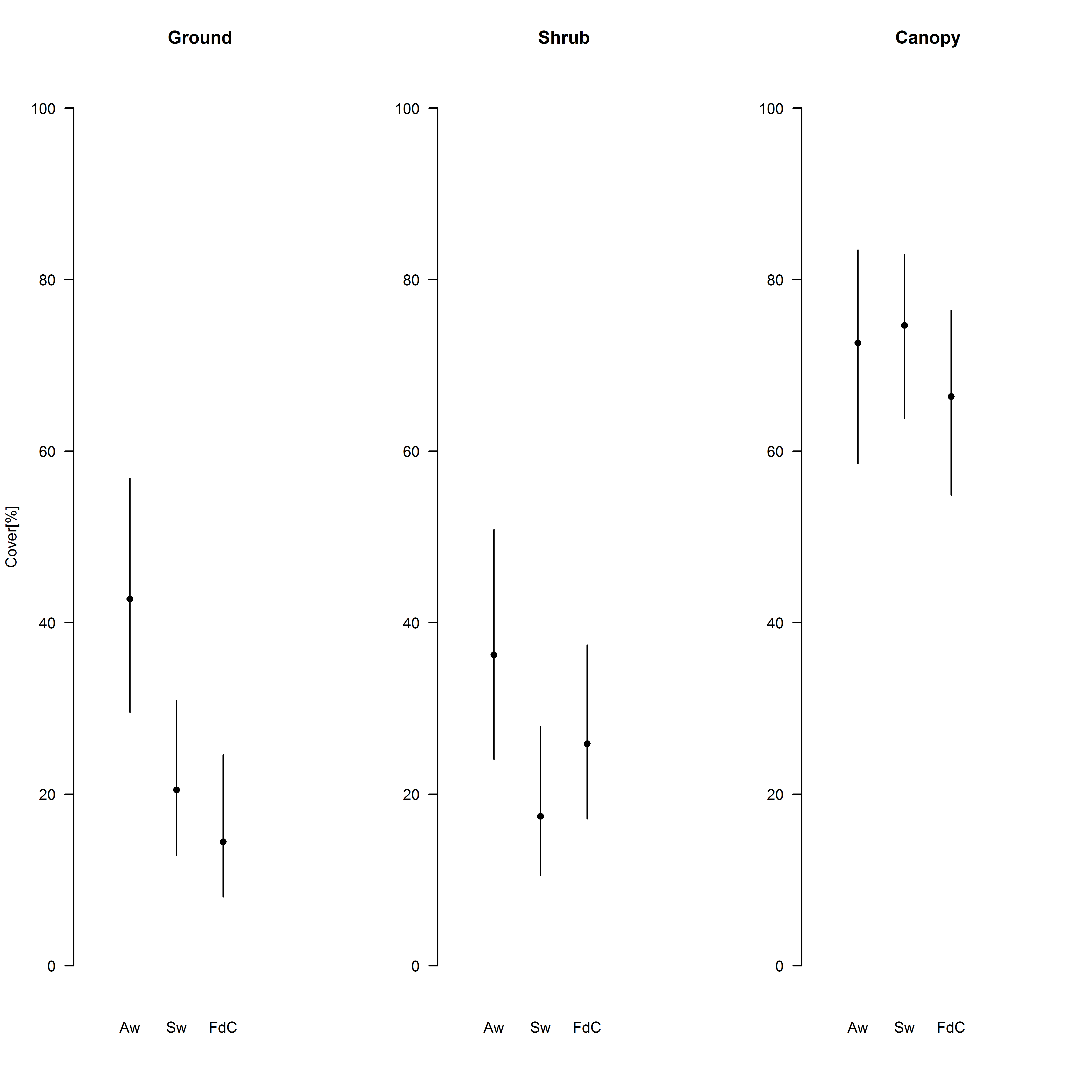

Supplement: Supplemental Information 1 — Note that vegetation measures were taken within the framework of a different study and thus do not entirely coincide in season or exact locations within forest with the FID measures. Therefore we could not directly include these measures in the model, but they still show that the forests were comparable in terms of habitat structure. There are differences in ground cover between certain forests. However, these differences are not likely to affect FIDs since our FID measures are taken in early spring (non-foliated season) and FIDs are usually more affected by higher-layers of vegetation (i.e. shrub and canopy layers). Aw: Allschwilerwald, Sw: Sihlwald, FdC: Forêt de Chaux. [file peerj-06-5093-s001.png]
